# Thrombosis in inflammatory bowel diseases: what’s the link?

**DOI:** 10.1186/s12959-015-0044-2

**Published:** 2015-04-02

**Authors:** Martina Giannotta, Gherardo Tapete, Giacomo Emmi, Elena Silvestri, Monica Milla

**Affiliations:** Gastroenterology Department, AOU Careggi Regional Referral Center for Inflammatory Bowel Disease, Florence, Italy; Department of Experimental and Clinical Medicine, University of Florence and Patologia Medica Unit, AOU Careggi, Florence, Italy

**Keywords:** Inflammatory bowel disease, Ulcerative colitis, Crohn’s disease, Thrombosis

## Abstract

Inflammatory bowel disease affects more than 2 million people in Europe, with almost 20% of patients being diagnosed in pediatric age. Patients with inflammatory bowel disease are at increased risk of thromboembolic complications which may affect patients’ morbidity and mortality. The risk of the most common thromboembolic events, such as deep venous thrombosis and pulmonary embolism, are estimated to be three-fold increased compared to controls, but many other districts can be affected. Moreover, patients with ulcerative colitis and Crohn’s disease experience thromboembolic events at a younger age compared to general population. Many factors have been investigated as determinants of the pro-thrombotic tendency such as acquired risk factors or genetic and immune abnormalities, but a unique cause has not been found. Many efforts have been focused on the study of abnormalities in the coagulation cascade, its natural inhibitors and the fibrinolytic system components and both quantitative and qualitative alterations have been demonstrated. Recently the role of platelets and microvascular endothelium has been reviewed, as the possible link between the inflammatory and hemostatic process.

## Introduction

Inflammatory bowel disease (IBD), whose major forms are ulcerative colitis (UC) and Crohn’s disease (CD), is a chronic inflammatory condition characterized by local and systemic inflammation predominantly affecting the gastrointestinal tract but that may be associated to numerous extra-intestinal manifestations including thrombosis. IBD affects more than 2 million people in Europe, with almost 20% of patients being diagnosed in pediatric age; in Italy over 200 thousands patients are estimated to suffer from such diseases.

The association between IBD and venous thromboembolism (VTE) was first described in 1936 by Bargen et al., who observed 18 patients with VTE among over 1000 IBD patients followed at the Mayo Clinic [1]. Since then, several publications reported an increased risk of thromboembolic events (TE) affecting both the venous and arterial district in UC and CD patients. This condition seems to be a characteristic of IBD, not simply of inflammatory or intestinal chronic disease, because it has not been demonstrated in other conditions such as rheumatoid arthritis or coeliac disease [2,3]. The overall incidence of VTE in IBD patients is estimated to be 1%-8% [2-4] although necroscopy studies report higher rates, approximately 40% [5-8]. No significant difference was found in the risk of VTE between UC and CD patients. The risk of VTE in lower extremities and pulmonary embolism in IBD patients is reported to be 3-fold increased compared to the general population [3,9,10] even after correction for known pro-thrombotic factors [6,7]. Other venous districts may occasionally be involved as the cerebral [11], hepatic and portal [12], retinal [13] and mesenteric veins [14]. Arterial thrombosis occur less frequently but has been described especially in cerebral, retinal and limbs arteries [15-17]. Some authors also reported an increased incidence of coronary [18] and aortic [19] thrombotic involvement in young IBD patients. IBD patients experiencing a VTE episode were generally younger compared to the general population [20], with the young age of first VTE being associated with a higher risk of recurrence [21]. The overall mortality rate per episode is estimated to be as high as 25% [9]. The risk of TE is estimated to be almost three times higher in males than in females [21]. Some authors reported an increased risk of TE in pregnant IBD women when compared with the non-IBD obstetric population [22,23], but others did not [24]. Generally most of TE occur during active disease, even if a high rate of thrombosis (almost one third) is reported during remission and well controlled disease, supporting the hypothesis of a greater pro-thrombotic tendency in IBD independent of disease activity [25,26]. The incidence of TE also correlates with the extent of the disease, i.e. especially pancolonic involvement in UC or colonic involvement in CD [27,28], and with the presence of complications such as fistulas, abscesses or strictures [28].

The pathogenesis of thrombosis in IBD is complex and not fully explained. It is thought to be multifactorial as no consistent unifying etiology has been found yet. Numerous investigations have been conducted on major pro-thrombotic genetic predispositions and IBD, but no significant association has been found to explain the increased VTE risk both in UC and CD patients [29-31]. Many reports tried to explain the increased risk of TE in IBD focusing on the different components of the coagulation cascade demonstrating both qualitative and quantitative abnormalities in procoagulant, anticoagulant and fibrinolytic factors, although others could not find any identifiable reason in approximately half IBD patients which developed TE [32]. Finally, interesting results have been found observing that in patients with inherited bleeding disorders (hemophilia A and B, von Willebrand disease) UC and CD occurred less frequently than in general population, giving indirect evidence that vascular thrombosis may be involved in IBD pathogenesis and in the inflammatory process [33].

### Acquired risks factors for thrombosis in IBD

Many acquired factors may affect hemostatic system and contribute to the pathophysiology of VTE in IBD patients. They include fluid depletion, prolonged immobilization, surgery, the use of central venous catheters, steroid therapy, oral contraceptives or hormone replacement therapy, cigarette smoking and vitamin deficiency leading to hyperhomocysteinemia [34,35]. In particular, hyperhomocysteinemia is known to be and independent risk factor for both atherosclerotic vascular disease and VTE [36], even if recently it has been considered more as a risk “marker” than as a true risk factor for thrombosis. A recent study reported significantly higher homocysteine plasma levels in UC and CD patients compared to controls, but the prevalence of hyperhomocysteinemia was not different within IBD patients which did or did not experience TE [37]. It is thought that folate deficiency related to the use of some medications (i.e. methotrexate or sulfasalzine) may lead to hyperhomocysteinemia in these patients although B6 and B12 vitamin or genetic mutations may also contribute to TE [36]. Acquired risk factors for thrombosis in IBD are summarized in Table 1.

**Table 1:** **Acquired risks factors for thrombosis in IBD**

1	Fluid depletion
2	Surgery
3	Central venous catheters
4	Immobilization
5	Steriod therapy
6	Oral contractive/hormone replacement theraphy
7	Vitamine defiency
8	Hyperomocystenemia
9	Cigarette smoking

### Genetic risks factors for thrombosis in IBD

The most frequent causes of inherited thrombophilia in the general population are the prothrombin G20210A polymorphismfactor and V Leiden, a genetic polymorphism that makes activated factor V relatively resistant to degradation by activated protein C. Less relevant is the C677T polymorphism of methylenetetrahydrofolate reductase (MTHFR) gene that favors hyperhomocysteinemia. These genetic mutations have been studied in IBD population but no significant difference has been found in their incidence in IBD patients when compared to health controls, nor in IBD patients that experienced or not TE [38,39]. An inherited polymorphism (Val34Leu) of factor XIII which protects against thrombosis has been evaluated in IBD patients and no difference has been found versus the general population [40,41]. Finally protein C, protein S and antithrombin deficiencies have also been investigated and they seem to have no increased prevalence among IBD patients independently of previous history of TE [36].

In conclusion, in spite of the big effort in terms of numerous genetic studies performed, no significant association has been demonstrated to date between genetic factors cause of hyper-coagulability and IBD. Thus, genetics of thrombophilia does not explain the increased TE risk in CD and UC patients, suggesting a most relevant role of acquired factors.

### Abnormalities of the coagulation cascade in IBD

**Figure 1:**
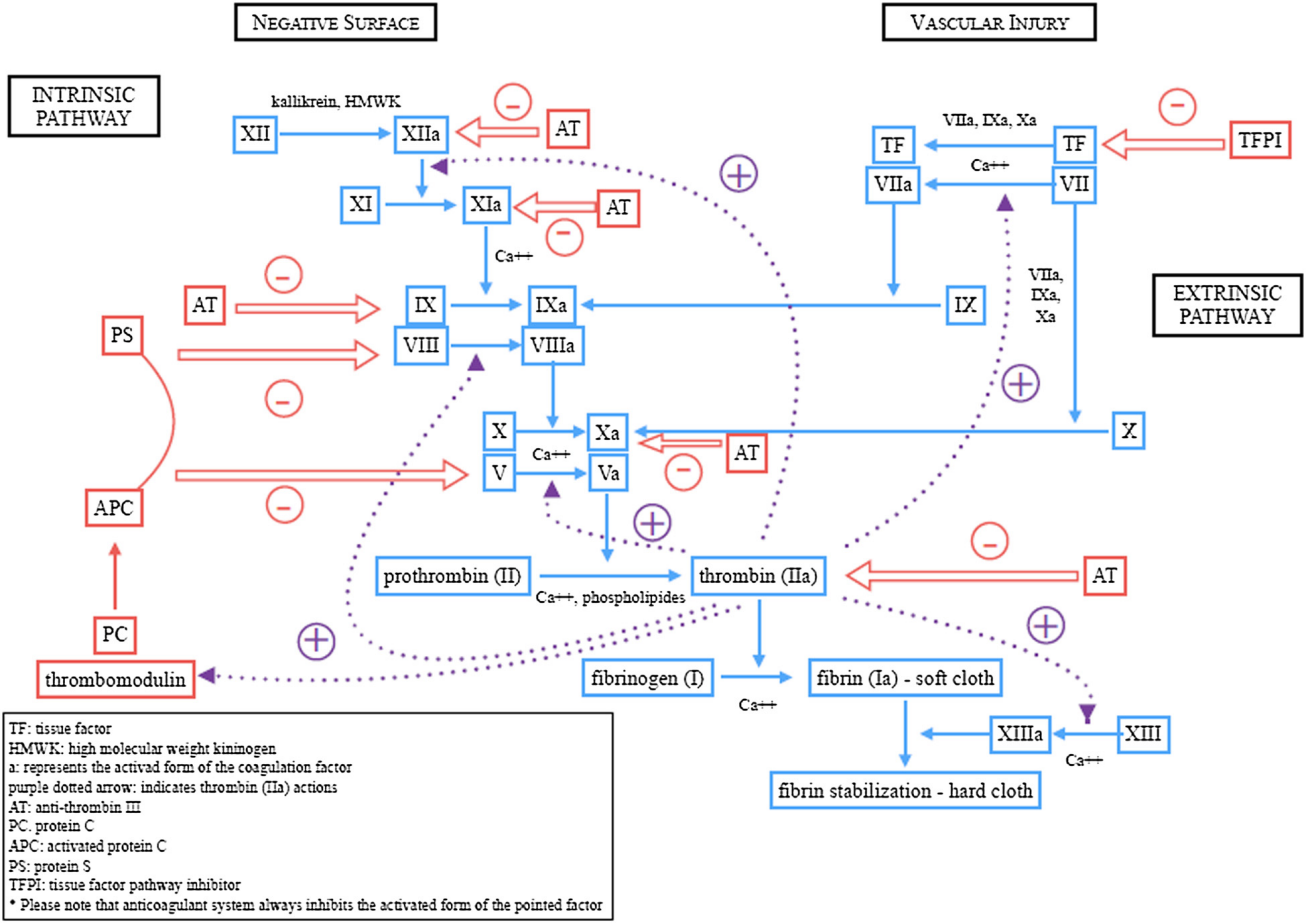
**Schematic representation of coagulative cascade and of natural anticoagulant system.**

The coagulation cascade is a series of enzymatic conversions ending in the formation of thrombin which converts fibrinogen (plasma soluble precursor) in fibrin (insoluble fibrous protein). The cascade has classically been divided into two branches: the extrinsic pathway initiated by tissue damage with exposition of tissue factor (TF) to blood, relevant for most conditions of clotting activation, and the intrinsic pathway which plays a role mainly in the mechanisms of amplification. The two ways converge at the point where factor X is activated, eventually leading to prothrombin cleavage and thrombin formation. A schematic representation of coagulation cascade and natural anticoagulant system is provided in Figure 1.

Several studies, in UC and CD patients, reported both quantitative and qualitative alterations of many coagulation enzymes, some of which are also considered acute-phase reactants. This includes elevation of circulating fibrinogen, prothrombin, and factors V (FV), VII (FVII), VIII (FVIII), X (FX), XI (FXI) and XII (FXII) [3,42-47]. Moreover, many other alterations reflecting a state of hyper-coagulability have been reported in IBD patients, such as elevation of prothrombin factor 1 + 2 (side-products of prothrombin cleavage), thrombin-antithrombin complex (TAT), fibrinopeptide A (FPA) and B (FPB) and decreased factor XIII (FXIII) levels [4,42,43,45,47,48]. These findings are consistent with an activation, which may sometimes be subclinical, of coagulation system in IBD. It is still debated if this condition is just a consequence of the inflammatory state or a feature of the intestinal disease per se regardless of disease activity [43,49]. In particular, decreased levels of FXIII, which is involved in the cross-linking of fibrin, have been reported mostly during active disease but FXIII mucosal deposits have been found both in affected and macroscopically normal bowel samples [43,49]. This is thought to reflect FXIII chronic consumption because of the increased formation of microthrombi in the vessels of inflamed enteric mucosa, or the repair of injured tissue [50,51]. Another mechanism that may be implicated in generating the pro-thrombotic state in IBD patients is thought to involve the cell components known as “microparticles” which are vesicles of cell membrane - mainly derived from platelets - released by cells, when activated, or during apoptosis. Microparticles have procoagulant properties because of the expression of TF on their surface and are implicated in modulation of endothelial activity and in inflammatory processes [52]. The circulating concentration of microparticles has been found to be elevated in IBD patients during active phases of the disease, suggesting a potential role in the procoagulant tendency [53]. Abnormalities of coagulation cascade in IBD are summarized in Table 2.

**Table 2:** **Abnormalities in coagulation, anticoagulation and fibrinolytic system in IBD**

**Coagulation factors**	**Fibrinolytic factors**	**Plasma coagulation inhibotors**
↑ Fibrinogen	↓ tPA	↓ AT III
↑ Prothrombin	↑ PAI-1	↓ TFPI
↑ Factors: Va, VIIa, VIIIa, Xa,XIa, XIIa	↑ TAFI	Conflicting data about PS and PC
↑ Prothrombin factors 1+2		
↑ Thrombin-antithrombin III complex (TAT)		
↑ Fibrinopeptide A and B		
↑ Microparticles		
↓ Factor XIII		

### Abnormalities of the natural coagulation inhibitors in IBD

A pro-thrombotic condition may result from a decrease in natural anticoagulant factors. The system of natural coagulation inhibitors is composed by antithrombin (AT), protein C (PC) pathway and TF pathway inhibitor (TFPI). AT is a physiological inhibitor of thrombin and FIXa, FXa, FXIa and FXIIa and its activation is triggered by heparin secreted by mast cells or exogenously administered. TFPI is a protein produced by the vascular endothelium and by megakaryocytes, and is an inhibitor of the TF-depending coagulation cascade and a raker of endothelial damage [54,55]. The PC pathway is a complex system which also involves protein S (PS) and several membrane receptors such as TM and endothelial protein C receptor (EPCR). PC is activated, especially if bounded to the EPRC, by the complex thrombin-TM. Once activated, in the presence of the cofactor PS, PC is able to inactivate FVIIIa and FVa and also has pro-fibrinolytic properties by binding PAI-1 [54,56]. See Figure 1 for schematic representation of coagulation cascade and natural anticoagulant system.

Data about the plasma concentration of natural coagulation inhibitors in IBD patients are conflicting: some authors reported no differences in PS [3,57,58] and AT [49,59] levels between IBD patients and controls, whereas others described a decrease in AT, TFPI, PC and PS in active *vs* inactive disease or *vs* controls [43,56,57,60]; finally some others found higher levels of PC and PS in IBD patients *vs* controls [43]. Some studies reported higher concentrations of TM in active CD and UC [61]. It is possible that changes in systemic levels of these factors do not necessarily reflect the local loss of inhibition of coagulation occurring within the enteric mucosa, where a loss of function of natural anticoagulants may occur [56].

Which seems important to remark is that there is a growing body of evidence that AT and TM are implicated in the inflammatory process. AT, when present at high concentrations [33], has been demonstrated to reduce the inflammatory response by decreasing leukocyte adhesion (by reducing expression of CD11b/CD18 cell surface receptors), TF and IL6 expression in monocytes and endothelium [55]. Furthermore AT seems to increase endothelial prostacyclin formation and inhibits endothelial pro-inflammatory mediators production by decreasing nuclear factor (NFkB) activation [54,62-64]. TM also seems to affect the course of inflammatory process, apoptosis and endothelial barrier integrity [54,55] and it has been suggested that increased TM levels may be associated with anti-inflammatory properties [61]. Abnormalities of coagulation inhibitors in IBD are summarized in Table 2.

### Abnormalities of the fibrinolytic system in IBD

**Figure 2:**
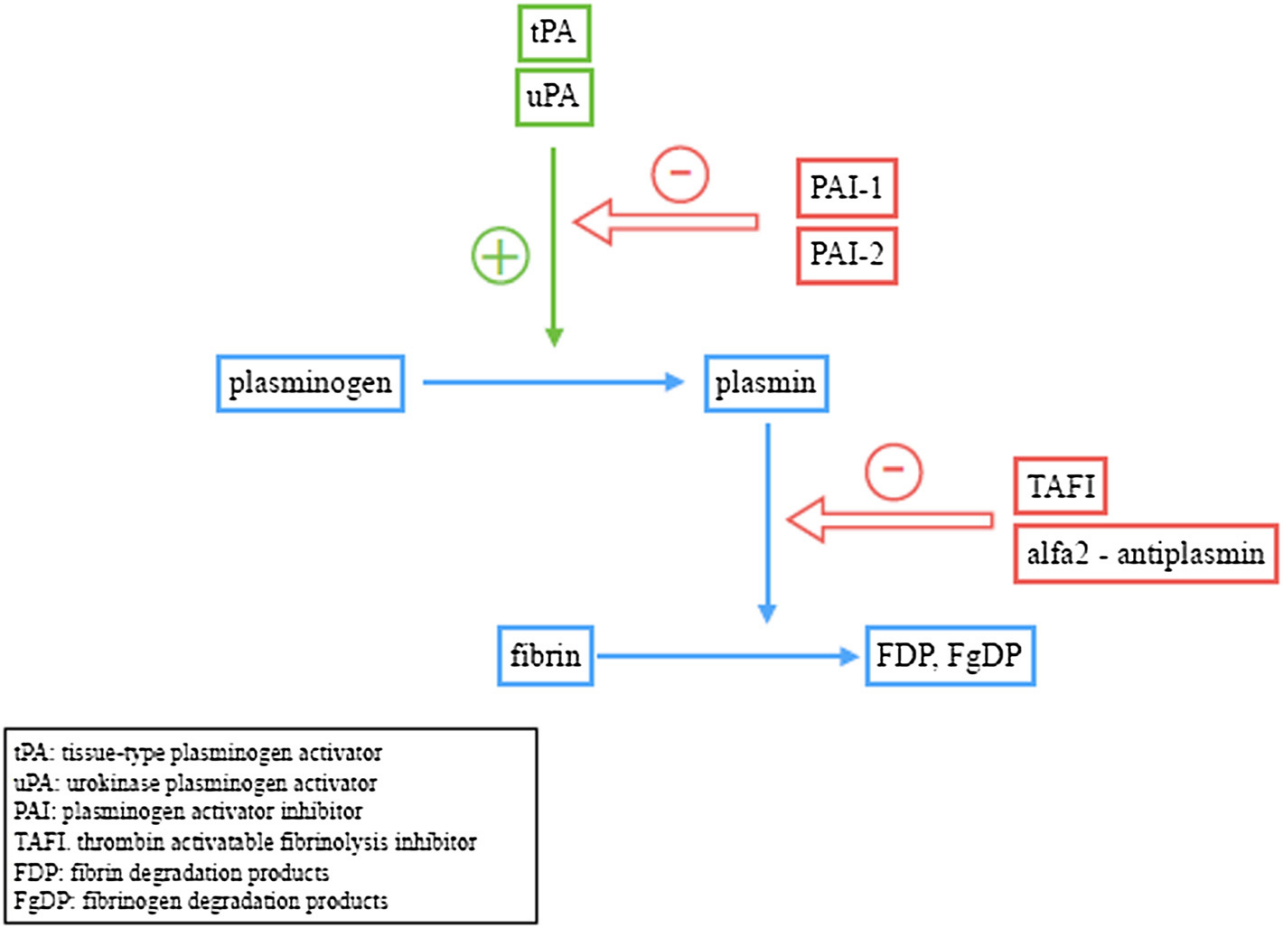
**Schematic representation of the fibrinolytic system.**

Normally the fibrinolytic system allows fibrin clot removal by plasmin activity which also inhibits several coagulation factors. Disturbances in the fibrinolysis may be associated with hyper or hypo-coagulability. The two major activators of plasminogen to plasmin are urokinase plasminogen activator (uPA) and tissue plasminogen activator (tPA). The latter, which is released in plasma by endothelial cells, is the most potent activator of plasminogen and consequently the main regulator of fibrinolysis. Its affinity to plasminogen is enhanced when bound to a fibrin surface thus restricting fibrinolysis to the site of clot [65,66]. The fibrinolytic system has potent inhibitors such as plasminogen activator inhibitor 1 (PAI-1) that inhibits tPA activity in plasma [67], alpha2-antiplasmin which inhibits plasmin activity in plasma [68] and thrombin-activatable fibrinolysis inhibitor (TAFI) which is responsible for the removal of carboxyl-terminal lysine residues from partially degraded fibrin, so decreasing the binding of tPA and plasminogen to the clot and consequently decreasing fibrinolysis, and is also a potent plasmin generation inhibitor. TAFI is activated by thrombin, thrombin-TM complex and plasmin thus being the direct link between coagulation and fibrinolytic systems [69]. See Figure 2 for schematic representation of the fibrinolytic system.

The circulating concentration of the fibrinolytic system’s factors is impaired in UC and CD patients and favors the pro-thrombotic mechanisms. In IBD patients a reduction in fibrinolysis activators (such as tPA) and an increase in inhibitors (such as PAI-1 and TAFI) has been described, inducing a reduced activity of the system [49,70,71]. Recently the activated form of TAFI (TAFIa) has been investigated because of its potential anti-inflammatory properties with recognition of its ability to inactivate anaphylotoxines, C3a, C5a and other pro-inflammatory mediators [72,73]. Moreover a significant correlation has been found in IBD patients between TAFIa, disease activity and inflammatory markers such as CRP, fibrinogen and platelets [73]. Abnormalities of the fibrinolytic system in IBD are summarized in Table 2.

### Abnormalities of the endothelium in IBD

Endothelium plays a central role in contributing to the inflammatory process by regulating leukocytes adhesion and transmigration and by the production of cytokines and chemokines. Furthermore it is responsible for the regulation of platelet adhesion and activation, and for the regulation of blood coagulation [56]. Endothelial disfunction has been demonstrated in IBD and markers of its damage such as von Willebrand factor (vWF), EPCR and TM are increased in IBD patients serum and seem to correlate with disease activity [74-77]. The knot seems to be an imbalance between nitric oxide (NO), a vasodilator and anti-aggregant agent, and reactive oxygen species (ROS) formation in the inflamed endothelium [36]. In fact NO production seems to be impaired in chronic inflamed IBD endothelium both from NO synthase 2 (NOS2) deficient transcription [78] and from induction of NOS competitor arginase by inflammatory citokynes such as IL2 and TNF-alpha [79]. On the other hand, an increased production of ROS has been demonstrated within the inflamed endothelium and it contributes to oxidative stress in vWF molecules leading to the accumulation of ultra-large vWF multimers - because of a reduced sensitivity to ADAMTS-13 proteolytic activity - which are most hemostatically active and favor platelet adhesion and aggregation [36,80].

Endothelium plays a key role in inflammation due to its ability to control the amount and type of leukocytes that transmigrate to the interstitial space and to regulate vascular tone and platelet adhesion and aggregation, thus it directly affects the hemostatic system potentially favoring thrombosis [56]. Which still remains unclear is if the vascular involvement in IBD is a pathogenetic factor or a consequence, inducing amplification of local and systemic inflammation [81,82].

### Abnormalities of platelets in IBD

In IBD patients there are quantitative, morphological and qualitative alterations in platelet characteristic. Thrombocytosis and IBD have been first correlated in 1968 [83] and it is now well established that trombocytosis is related to disease activity and severity [84]. Thrombocytosis is considered a non specific response to inflammation which may occur in chronic inflammatory conditions other than IBD [25], but it has also been proposed that thrombocytosis in UC and CD may reflect an aberration in thrombopoiesis induced by greater plasma levels of thrombopoietin and IL6, which are involved in megakaryocytes maturation process [85]. On the other hand, platelets in IBD patients have smaller mean corpuscular volume (MCV) than in controls [86], and platelet MCV seems to be smaller during active phases of the disease as compared to remission [56]. MCV has also been demonstrated to be inversely proportional to some inflammation markers levels such as C-reactive protein (CRP) and erythrocyte sedimentation rate (ESR) so that this has been proposed as a marker of disease activity [87]. It has been hypothesized that during active disease platelet agglomerates (platelet-platelet, platelet-leukocyte, platelet-endothelium) - that are found to be elevated in IBD patients [88] - mainly involve younger platelets with a higher MCV and consequently that would relatively increase the circulating amount of older and smaller platelets [89]. Moreover, platelets in IBD seem to circulate in a chronic activated status and to be more reactive and more sensitive to activation induced by pro-aggregating agents. First of all, platelets have been demonstrated to aggregate in vitro in over 30% of IBD patients, independently of disease severity, compared to none of the healthy controls [90]. This was thought to be a consequence of the inflammatory condition but it has subsequently been demonstrated that platelet aggregates are found in IBD patients but not in other inflammatory diseases, thus being a specific characteristic of IBD [91]. The same author found an increase in surface and serum platelet activation markers such as P-selectin, GP53 and beta-thromboglobulin (beta-TG), whose increase was independent from disease activity so suggesting that once stimulated, platelets may remain chronically activated even during remission phases of the disease [25,91]. In the recent years another piece has come to partially complete the picture. In fact, in CD and UC patients high values - up to 4 times greater than healthy controls - of the surface CD40 ligand (CD40L), an activation markers that allows platelets to interact with a broad of immune and non immune cells with pro inflammatory consequences [25,92], and that acts as an inducer of the TF mediated coagulation cascade [36] have been detected. Together with its increase on the platelet surface, even the soluble form of CD40L (sCD40L) in IBD patients serum is increased - almost 15 fold compared to controls - as released by such platelets [93]. High levels of sCD40L have been associated with an increased risk of TE [55]. Others reported that in vitro activated platelets may directly increase CD40L expression in intestinal endothelial cells favoring their interaction with numerous immune cells and sustaining chronic inflammation [94]. Recent data reported an increased expression of CD40/CD40L in the intestinal epithelial cells, in particular in samples from inflamed ileal and colonic mucosa from CD and UC patients, whereas that increase was not found in uninvolved intestinal segments [95]. This finding provide, for the first time, a piece of evidence of the interaction between activated platelets and IBD affected intestinal mucosa via the CD40/CD40L pathway [56]. That leads to a key point: platelets could themselves act as inflammatory cells and enhance the inflammatory process in IBD mucosa. One of the first suggestion of the platelet role in intestinal inflammation came from the finding of capillary microthrombi in the mucosa of IBD patients, independently of the severity of inflammation. Those findings were consistently absent in healthy subjects [96]. It has then been suggested that platelet activation occurs in the intestinal mucosa because of the finding of greater platelet aggregates in the mesenteric blood of CD patients [97]. This process has recently been reproduced in vitro using human intestinal microvascular endothelial cells (HIMEC) exposed to IL-1beta to mimic IBD endothelial changes [25,93]. Activated CD40L positive platelets are then thought to enhance themselves intestinal inflammation by the interaction with CD40 positive microvascular endothelium in the intestinal mucosa. That is thought to be the trigger to up-regulation of endothelial IL8 production and adhesion molecule expression (as ICAM-1 and VCAM-1) on the endothelium surface, leading to inflammatory cell, specifically T-cell, recruitment and inflammatory response amplification [25].

### The role of inflammation

Inflammation and coagulation are two crucial systems that are in balance and constantly influence each other [25]. The impact of inflammation on coagulation has been confirmed by several experimental studies showing that inflammatory mechanism shift the hemostatic balance to favor the activation of coagulation [36,54] which, in turn, can also sustain inflammation promoting a vicious circle between chronic inflammation and thrombosis [25]. We have seen above how platelets, endothelium and many components of the coagulation cascade, fibrinolytic system and natural anticoagulant inhibitors are directly involved in the inflammatory process but other considerations need to be done. Many studies reported that tumor necrosis factor alpha (TNF-alpha), CD40L and CRP are able to induce the expression of TF on the leucocytes cell surface, so promoting activation of the intrinsic coagulation pathway [98-100]. On the other hand, natural anticoagulant pathways as the PC pathway and the heparin-AT pathway have been demonstrated to be down-regulated by inflammatory citokynes as TNF-alpha and IL1beta [101,102]. Furthermore, CRP has been shown to inhibit TFPI [103] and tPA [104] and to increase the expression of PAI-1 [105] leading to a more procoagulant tendency. Another inflammatory citokyne, IL6, has been shown to have a pro-thrombotic activity by increasing platelet production and enhancing thrombin formation in concert with TNF-alpha [106,107]. Another trigger to the procoagulant profile by TNF-alpha is the down regulation of the expression of anticoagulant TM and EPCR [108]. Recently, homocysteine has been shown to participate to microvascular inflammation by triggering, together with TNF-alpha, the expression of VCAM-1 and MCP-1 on endothelial surface thus leading to an enhanced capacity to recruit T cells and monocytes [109].

### Abnormalities of the immune system in IBD

Anti-phospholipid antibodies (APLA) are a group of pro-thrombotic antibodies including lupus anticoagulant (LAC), anti-cardiolipin antibodies (aCL) and anti-beta-2-glicoprotein-I (anti-beta-2-GPI). APLA may be associated with both venous and arterial thrombosis. IBD patients seem to have higher rates of aCL and anti-beta-2-GPI positivity - with an incidence of 20%-30% for the first and 9% for the latter - compared to general population, but the real association with thrombosis in IBD is still unclear [110,111]. In fact, LAC, aCL and anti-beta-2-GPI levels were similar in IBD patients with or without history of VTE [36,110].

In IBD patients anti-PS antibodies have also been detected, which may cause a reduction in the natural anticoagulant potential but there is still not enough evidence that they play any role in thrombotic risk [112].

## Conclusions

Thrombotic mechanism in IBD patients is complex, multifactorial and not completely understood. Acquired risk factors for thrombosis, often present in IBD patients, only partially explain the increased thrombotic risk in this particular population. No relevant association has been found regarding genetic pro-thrombotic risk factors in UC and CD patients. Moreover, many quantitative and qualitative alterations in single components of cascade factors, fibrinolytic system and natural anticoagulants has been found, but none of them was sufficient per se to explain the increased thromboembolic risk. What seems to be the case is that maintaining the pro-thrombotic tendency in this context is multifactorial, thus coagulation components and their inhibitors, as well as hemostatic relevant cells as platelets and endothelial cells, interact in a context of inflamed mucosa that is chronically activated and contributes to maintain chronic inflammation as well. In turn, inflammatory molecules and cytokines have been demonstrated to imbalance the haemostatic system towards hyper-coagulability. The two complex mechanisms of inflammation and coagulation deeply interact in IBD mucosa amplifying and potentiating each other.
